# The Association of Air Pollution With the Patients’ Visits to the Department of Respiratory Diseases

**DOI:** 10.14740/jocmr2174e

**Published:** 2015-05-08

**Authors:** Yanran Cai, Yumeng Shao, Chenggang Wang

**Affiliations:** a Basic Medical College of Shandong University of Traditional Chinese Medicine, Jinan, Shandong, China

**Keywords:** Daily number of patients, Particulate matter 2.5, Outpatient department of respiratory diseases, Generalized additive model

## Abstract

**Background:**

The objective of this study was to evaluate the impact of air particulate matter 2.5 (PM2.5) on the daily number of patients’ visits to the Department of Respiratory Diseases in a local general hospital.

**Methods:**

The number of patients in outpatient department of respiratory diseases (ODRD) in a general hospital of Jinan, China, the air quality and meteorological data were collected for 1 year. By controlling the confounding factors such as “day of the week” effects and the meteorological factors, the generalized additive Poisson regression analysis was conducted to evaluate the impact of PM2.5 on the number of patients’ visits to the ODRD.

**Results:**

Within two consecutive days, if the cumulative PM2.5 was less than 200 µg/m^3^, the daily number of patients in the ODRD did not increase significantly; however, it increased dramatically when the concentration of PM2.5 particles reached the range between 200 and 400 µg/m^3^.

**Conclusion:**

There is a non-linear relationship between the concentration of atmospheric PM2.5 particles and the daily number of patients in the ODRD.

## Introduction

Suspended particles in the air can be divided into large particulate matter (PM) (diameter between 11 and 100 µm) and inhalable PM (diameter smaller than or equal to 10 µm, known as PM10). PM10 is divided into coarse PM (diameter between 2.5 and 10 µm) and fine PM (diameter less than or equal to 2.5, PM2.5). PM2.5 is defined as particles that are able to enter the lungs. Considerable evidence has demonstrated that atmospheric particles can have a tremendous impact on human health, the smaller the particle size, the greater the harm it may cause. The harm caused by fine particles of dust haze weather on human health is even greater than by a sand storm, as PM2.5 particles may contain a large number of toxic and harmful substances, stay longer in the atmosphere, convey long distance, and are easy to trigger asthma, bronchitis and other diseases [1]. PM2.5 particles can also be the carrier of viruses and bacteria to spread infectious diseases of the respiratory tract. In recent years, as air pollution in Jinan area is severely worse, PM2.5 is the main cause of haze weather, and its harm to environment and human health should not be neglected. The quantitative evaluation of health hazards of PM has become the research focus of WHO, the European Union and other international organizations. WHO pointed out that the PM2.5 higher than 25 µg/m^3^ is harmful to human health [2]. Evidence showed that exposure to fine particulate air pollution was related to prevalent anxiety [3]. Long-term exposure to air pollution increased the incidence of blood hypertension in elderly [4, 5]. This study intended to evaluate the impact of PM2.5 level on the patients’ visits to the Department of Respiratory Diseases in a general hospital in Jinan City, China. Because their relation may be not in simple linear, we used daily number of patients in the outpatient department of respiratory diseases (ODRD), time sequence method, and generalized additive models (GAM) to analyze the impact of PM2.5 level on the number of patients’ visits in the ODRD. We expected that these data may provide scientific evidences for the prediction of adverse effect of pollutants on human health.

## Materials and Methods

### Data collection

The database was the daily number of patients’ visits to the ODRD of the Jinan Hospital of Traditional Chinese Medicine, an affiliated Hospital of Shandong University of Traditional Chinese Medicine in Jinan City, China, from November 1, 2013 to October 31, 2014. According to the database, patients who were local residents within a distance of 18 km from the hospital were included in this study. Patients’ statistics were made according to the international classification of diseases 10th edition (ICD-10), and patients with respiratory diseases were encoded from J00 to J99. This study was approved by the Research Ethics Board (REB) of Shandong University of Traditional Chinese Medicine.

### PM2.5 data source

The average levels of daily PM2.5, including sulfur dioxide (SO_2_), nitrogen dioxide (NO_2_), carbon monoxide (CO) and other air quality data, as well as daily temperature range, and other meteorological data were from the Jinan Meteorological Bureau website (http://www.tianqihoubao.com) between November 1, 2013 and October 31, 2014.

### Statistical analysis

#### Determination of the independent variables

In this study, the relationships between SO_2_, NO_2_ and CO tended to be co-linearity (correlation coefficient between any two was greater than 0.74). In order to improve the accuracy of model estimation, we used the principal component analysis to extract the common factor (factor 1) from the three variables (the cumulative contribution rate was 86.1%) as the independent variable. The Pearson correlation coefficient between the daily lowest and highest temperatures was 0.97, whereas the lowest temperature was used as the independent variable. Cross-correlation analysis was used to explore the lag effect of PM2.5 on the response variable. Based on the cross-correlation coefficient and the cumulative exposure effect, the sum of PM2.5 values in two consecutive days (PM2.5td) was considered to be the independent variable. In order to prevent the effect of “day of the week”, week factor (week) was extracted as the independent variable in this study.

#### The choice of statistical models

**Figure 1:**
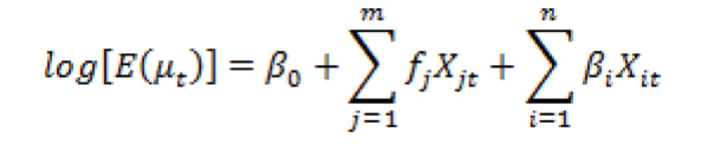
The generalized additive models formula.

The daily number of patients in the ODRD was defined as a response variable, which approximately conformed to Poisson distribution. Due to the possible non-linear relationship between the above variables and daily outpatient visits, the GAM was used to fit the data, and the connection function (link function) was logarithmic function. The formula is shown in Figure 1.

In the formula, E(µ_t_) is the expected number of patients on day t of the outpatient department, X_j_ is the independent variable that has non-linear relationship with the response variable, X_i_ is the independent variable that has non-linear relationship with the response variable, β_j_ is regression coefficient, and f_j_ is smoothing spline function. The above statistical analyses were performed using SAS 9.2 with the standard of small probability event α < 0.05.

## Results

### Descriptive analysis

**Figure 2:**
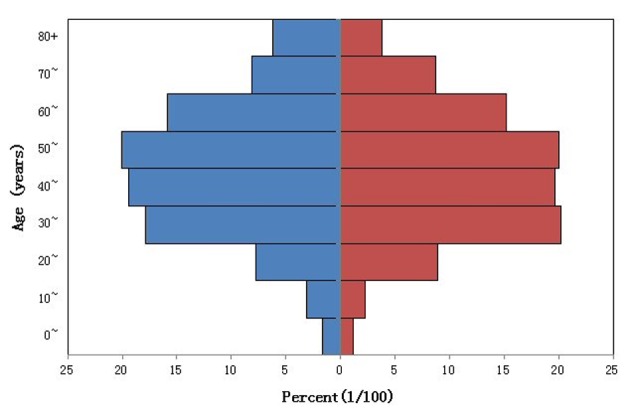
The age distribution of department of respiration patients. Blue: male; red: female.

The total number of patients in the ODRD was 30,837 during the study period, among whom 48.6% were male, and mean age was 49.14 years. The age distribution is shown in Figure 2. Diagnoses of patients were mainly divided into upper respiratory tract infection (23,408 cases, 76%), lower respiratory tract infection (5,817 cases, 19%), and the other (1,612 cases, 5%). The daily number of patients in the ODRD was at least 26 patients in Jinan Hospital of Traditional Chinese Medicine, and at most 369 patients. The average daily number of patients in the ODRD was 84.82 patients, while on Saturday or Sunday, it was about 0.89 times less than that on weekdays. The concentration of air pollutants and the minimum temperature of the descriptive analysis results are shown in Table 1. PM2.5td is less than 200 μg/m^3^ in 245 days, accounted for 67.6%, and less than 400 μg/m^3^ in 348 days, accounted for 95.6%.

**Table 1:** Concentration of Air Pollutants in Jinan City and the Minimum Temperature of the Descriptive Analysis During the Study Period

	Min	Max	Median	Four quartile range
T_min_ (°C)	-10.00	29.00	14.00	18.00
PM2.5 (μg/m^3^)	21.00	444.00	81.00	52.75
PM2.5td (μg/m^3^)	60.00	773.00	163.00	101.50
CO (mg/m^3^)	0.38	4.19	1.12	0.69
NO_2_ (μg/m^3^)	13.00	134.00	52.50	28.00
SO_2_ (μg/m^3^)	17.00	228.00	58.50	63.75

### Model fitting analysis

**Figure 3:**
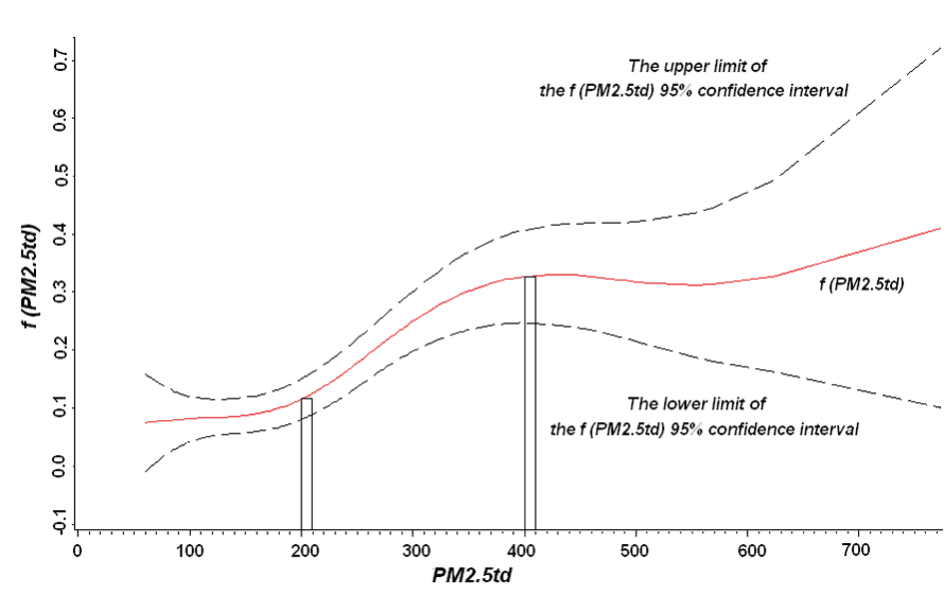
Non-linear effect chart between the sum of PM2.5 within 2 days (PM2.5td) in Jinan and the quantity of patients in the outpatient department of respiratory diseases (ODRD).

The fitting results were divided into parametric regression analysis, smoothing spline non-parametric analysis and analysis of variance, and the distribution is shown in Table 2-4. In Table 2, all independent variables were statistically analyzed to determine the parameter regression, and factor 1 was a risk factor. In Table 3, the non-parametric analysis of the smooth components showed that the generalized cross-validation (GCV) is smaller, and the degrees of freedom of two variables were about 4.2. The relationship between the sum of PM2.5td and the quantity of patients in the ODRD is shown in Figure 3, indicating that the fitting curve tends to be smooth; therefore the model fitting effect would be well accepted. Table 4 shows the non-parametric analysis of variance, to compare the whole model and the deviation which does not contain the variables. Only the minimum temperature and the PM2.5td were found statistically significant. Based on the coefficients in the model, the relative risk (RR) of the independent variable PM2.5td and the quantity of patients in the ODRD can be calculated according to the formula: RR = e^f(PM2.5td)^. Figure 2 shows a curve relationship more intuitively between the PM2.5td and the patients’ visits in the ODRD in Jinan. When the 2-day cumulative PM2.5 was less than 200 μg/m^3^, the patients’ visits to the ODRD did not change dramatically with the increase of PM2.5, and there was less change especially when PM2.5 was lower than 150 μg/m^3^. On the contrary, when PM2.5 reached 200 μg/m^3^, the patients’ visits to the ODRD increased significantly; however, once PM2.5 was higher than 400 μg/m^3^, the curve became flat.

**Table 2:** Regression Analysis of the Estimated Values of Model Parameters

Variables	Estimated values	SEM	t value	P value
Intercept	3.5811	0.0484	73.96	< 0.0001
Factor 1	0.0265	0.0084	3.16	0.0017
Week	0.0030	0.0002	17.32	< 0.0001
Linear (T_min_)	-0.0109	0.0007	-15.38	< 0.0001
Linear (PMtd)	0.0011	0.0001	14.49	< 0.0001

**Table 3:** Quantitative Analysis of Smooth Components in Non-Parameter Models

Smooth components	Smooth parameter	Degree of freedom	Generalized cross-validation (GCV)	No. of variables at different values
Spline (T_min_)	0.9982	4.2724	0.0497	40
Spline (PMtd)	0.9999	4.2320	0.0551	193

**Table 4:** Analysis of Variance of the Non-Parameter Model

Smooth components	Degree of freedom	Sum of square	χ^2^ value	P value
Spline (T_min_)	4.2724	312.93	48.57	< 0.0001
Spline (PMtd)	4.2320	99.69	15.47	0.0047

## Discussion

GAM is the extension of a generalized linear model (GLM), which can handle complex non-linear relationship between the response variables and the independent variables, and is widely applied in the environmental epidemiological study and other areas [6]. In terms of application of GAM, it is assumed that the function (f) can be added. To estimate the relationship between the response variable and independent variable, what to explain can be the variable itself, and also can be various smoothing functions of independent variables with flexibility [7]. GAMs are commonly used to study the effect of environmental pollution on health. In the smoothing spline function, how to determine degree of freedom may produce some impact on the fitting effect of the function. With increase of degree of freedom, the function fitting may be better; however, it would be able to result in a rough curve [6]. As the GCV is smaller in this study, and the curve is relatively smooth, good fitting results were obtained.

The relationship between PM2.5 and daily mortality in a meta-analysis conducted by Qian et al found that the health effects on the residents who were exposed to atmospheric PM2.5 pollution for a short term included mortality increase of severe illness and chronic diseases, deterioration of diseases of the respiratory system and cardiovascular system, the changes of pulmonary function and structure, and increase of incidence [8]. In an experimental study, rats were exposed to PM2.5; with increasing PM2.5 concentration, the activity of antioxidant enzymes (SOD, GSH-Px, CAT) and content of GSH in the heart, lung, and testis three organs appeared to reduce or decrease dramatically [9]. Sagai et al found that PM2.5 was able to produce reactive oxygen species (ROS), and generation of ROS was closely related to the injury of the respiratory and circulatory systems [10].

Our results showed that, when the 2-day cumulative PM2.5 was lower than 200 μg/m^3^, the number of patients’ visits to ODRD did not increase even if the PM2.5 concentration went up, especially when the concentration was lower than 150 μg/m^3^. When exposed to low concentration of PM2.5, most people may have adapted to the load or compensatory state. However, when the concentration exceeded 200 μg/m^3^, the patients’ visits to the ODRD increased rapidly, and health effect became more obvious. When higher than 400 μg/m^3^, the curve became flat, probably because the data of this study were only collected for 1 year. There were relatively few days when PM2.5 concentration was more than 400 μg/m^3^, thus the effect estimation was unstable.

After excluding the effect from the main atmospheric pollutants and temperature, the delay effect and cumulative effect of PM2.5 concentration on the respiratory system diseases were analyzed. There was a non-linear relationship between PM2.5 levels and the patients’ visits to the ODRD. This will provide basic information for air pollution prediction, early warning, and for future studies, and also provide a scientific basis for public health research. In this study, the results would be more reliable if the multi-factorial analysis was conducted, including the chemical composition of PM2.5 particles in the atmosphere and other meteorological data such as air pressure, air humidity.

This study focused on the most common diseases of the respiratory system such as pneumonia, bronchitis, and so on. Epidemiological studies have shown that haze weather mainly affects the respiratory system and cardiovascular system, exposure to a certain amount of PM2.5 can cause the number of visits for respiratory and cardiovascular diseases and inpatients increased [11]. Many studies showed that PM2.5 had more influence on human health and was more closely related to mortality than PM10 [12]. PM2.5 may come from a variety of sources of air pollution, as coal is continuously used as the main energy source in China; meanwhile, the number of motor vehicles increases dramatically, and PM2.5 pollution is becoming more and more serious so that the investigation on the impact of PM2.5 on pathogenesis of respiratory diseases is important, practical and has long-term significance. Based on the obvious harm of PM2.5 on human body, all relevant departments should take active measures, such as enhancing propaganda of the PM2.5-associated diseases to let people realize its harm, stipulating national standards of fine PM in the air, and reducing the production and release of fine PM. It would be able to reduce the related diseases’ prevalence and protect the people’s health to the maximum.
